# The Gastrointestinal Tract Microbiome and Potential Link to Alzheimer’s Disease

**DOI:** 10.3389/fneur.2014.00043

**Published:** 2014-04-04

**Authors:** James M. Hill, Surjyadipta Bhattacharjee, Aileen I. Pogue, Walter J. Lukiw

**Affiliations:** ^1^LSU Neuroscience Center, Louisiana State University Health Sciences Center, New Orleans, LA, USA; ^2^Department of Microbiology, Louisiana State University Health Sciences Center, New Orleans, LA, USA; ^3^Department of Ophthalmology, Louisiana State University Health Sciences Center, New Orleans, LA, USA; ^4^Alchem Biotek, Toronto, ON, Canada; ^5^Department of Neurology, Louisiana State University Health Sciences Center, New Orleans, LA, USA

**Keywords:** Alzheimer’s disease, microbiome, genetic complexity, evolution, Bacteroidetes, Firmicutes, BMAA, BDNF

Accumulating clinical- and scientific research-based evidence is driving our increased awareness of the significance of the human microbiome (HM) to the healthy and homeostatic operation of the human central nervous system (CNS). HM communities occupy several different but distinct microbial ecosystems on and within the human body, including nasal, oral, and otic cavities, the surface of the skin and the urogenital and the gastrointestinal (GI) tracts. The complex symbiotic inter-relationship between the GI-tract microbiome and its host is strongly influenced by diet and nutrition, and when optimized can be highly beneficial to food digestion, nutrient intake, and immune health (1–6). For example, dietary composition ultimately affects the structure, organization, function, and speciation of the HM occupying the GI tract, in part by supplying multiple substrates for microbial metabolism. Typical Western diets containing high fat–cholesterol, low amounts of soluble and insoluble fiber, and sugar- and salt-enrichment not only impart deleterious nutrition but also dietary constraints on the HM. This in turn impacts the supply of microbiome-generated molecules absorbed into the systemic circulation for transport into the extensive neurovasculature of the CNS. This short communication will focus on emerging ideas concerning the contribution of the GI-tract microbiome to human neurological disease with emphasis on Alzheimer’s disease (AD) wherever possible.

It is the HM of the GI tract that contains the largest reservoir of microbes in humans, containing about 10^14^ microorganisms from at least 1000 distinct microbial species, and outnumbering human somatic cells by about 100 to 1 (1, 7). The total HM has been estimated to encode about 4 × 10^6^ genes versus the ~26,600 genes of the human host, so again the quantity of HM genes outnumbers host genes in the order of about 150 to 1 (4). Of the 55 bacterial divisions currently identified, only two are prominent in mammalian GI-tract microbiota, including the anaerobic *Bacteroidetes* (~48%) and *Firmicutes* (~51%), with the remaining 1% of phylotypes distributed amongst the *Proteobacteria*, *Verrucomicrobia*, *Fusobacteria*, *Cyanobacteria*, *Actinobacteria*, and *Spirochetes*, with various species of fungi, protozoa, viruses, and other microorganisms making up the remainder (http://www.genome.gov/pages/research/sequencing/seqproposals/hgmiseq.pdf). Interestingly, microorganisms making up the smallest proportion of the HM seem to have a disproportionately large effect on host health and disease (see below). Of all GI-tract microbiota, bacterial densities of 10^11^–10^12^/ml are the highest recorded density in any known microbial ecosystem of any living organism (1, 4, 7–10). There is currently expanding interest in the ability of these high density GI-tract bacteria to influence host innate-immune, neuromodulatory-, and neurotransmission-functions (3, 4, 11–14). Established pathways of GI–CNS communication and mutualism currently include the autonomic nervous system (ANS), the enteric nervous system (ENS), the immune system, and the neuroendocrine system (15–21). Remarkably, neuronal signaling pathways along this bidirectional GI–CNS axis remain incompletely understood despite their important roles: (1) in coordinating metabolic-, nutritive-, and homeostatic-functions, and (2) in their functional disruption in chronic diseases such as anxiety, autoimmune-disease, diabetes, metabolic-syndrome, obesity, and stress-induced and progressive neuropsychiatric diseases including AD (3, 11, 12, 20, 22–24).

Here we list six specific, highly illustrative examples and recent insights into the interactive nature of the HM with a healthy, homeostatic CNS, and examples of a dysfunctional or altered HM contribution to the development of age-associated neurological disease:

(1) studies of the ENS in germ-free “*gnotobiotic*” mice, i.e., those missing their microbiome, indicate that commensal GI-tract microbiota are critically essential for membrane electrical characteristics, including ion fluxes, action potentials, and GI-tract sensory neuron excitability, thus providing a potential mechanistic link for the initial exchange of signaling information between the GI-tract microbiome and the ANS, ENS, CNS neuroimmune–neuroendocrine systems (4, 5, 20, 23, 25);
(2) GI-tract-abundant Gram-positive facultative anaerobic or microaerophilic *Lactobacillus*, and other *Bifidobacterium* (*Actinobacteria*) species such as *Lactobacillus brevis* and *Bifidobacterium dentium* are capable of metabolizing glutamate to produce gamma-amino butyric acid (GABA), the major *inhibitory* neurotransmitter in the human CNS (26). Increased GI-tract GABA appears to correlate with increased CNS GABA levels, but the systemic pathways that contribute to this gut–brain linkage require additional study (3, 26). In CNS dysfunctions in GABA-mediated neuromodulatory and neurotransmission functions have been linked to the development of anxiety, behavioral deficits, epilepsy, defects in synaptogenesis, depression, and cognitive impairment including AD (16, 17, 23, 27–29). Interestingly, epileptic activities including complex partial-seizures and non-convulsive seizures are commonly associated with AD, especially in its early stages, but the contribution of GI-tract microbiome to epileptiform events via GABA modulation is not well understood (30);
(3) the secreted, dimeric, 238 amino acid brain-derived neurotrophic factor (BDNF) essential in the maintenance and survival of neurons, has pleiotropic effects on neuronal development, differentiation, synaptogenesis, and the synaptic plasticity that underlies neuronal circuit formation and cognition, and has been found to be decreased in brains and serum from patients with anxiety, behavioral defects, schizophrenia, and AD (27, 31, 32). Interestingly, mice deficient in BDNF have altered development of GI-tract innervations including the vagus nerve, which normally serves as a major constitutive, modulatory communication pathway across the GI–CNS axis (33, 34). In experimental infection models known to lead to significant alterations in the microbiota profiles, BDNF expression was found to be reduced in the hippocampus and cortex of germ-free “*gnotobiotic*” mice, and the reduction in the expression of BDNF was found to specifically associate with increased anxiety and progressive cognitive dysfunction (20, 31, 32);
(4) glutamate is the most abundant *excitatory* neurotransmitter in the human CNS; the *N*-methyl-d-aspartate (NMDA) glutamate receptor, a CNS-enriched transmembrane sensor that regulates synaptic plasticity and cognition has some intriguing and potentially direct interactions with the HM; for example, the NMDA-, glutamate-targeting, glutathione-depleting, and oxidative-stress-inducing neurotoxin β-*N*-methylamino-l-alanine (BMAA), found elevated in the brains of patients with amyotrophic-lateral sclerosis (ALS), the Parkinson-dementia complex of Guam, and AD, has been hypothesized to be generated by *Cyanobacteria* of the GI-tract microbiome, and anxiety, stress, chronic intestinal inflammatory disease, or malnutrition may further induce BMAA generation to ultimately contribute to neurological dysfunction (13, 35). Interestingly, BMAA, a neurotoxic amino acid not normally incorporated into the polypeptide chains that constitute brain proteins, has been linked with intra-neuronal protein misfolding, a hallmark feature of the amyloid peptide-enriched senile plaque lesions, and resultant inflammatory neurodegeneration, that characterize AD, ALS, PD, and prion disease (21, 23, 36). These and other HM-resident *Cyanobacteria*-generated neurotoxins including saxitoxin and anatoxin-α may further contribute to neurological disease, especially over the course of aging when the intestinal epithelial barrier of the GI tract becomes significantly more permeable (13, 37);
(5) the HM not only secretes nutritive molecules, including essential vitamins of the B and K group, but also release molecular factors that may potentially modulate or alter systemic- and CNS-amyloidosis, CNS neurochemistry, and neurotransmission. For example, HM organisms widely utilize their own naturally secreted peptides and amyloids as structural materials, adhesion molecules, and neurotoxins that ultimately function in host auto-immunity and immune-protection. The specific contribution of the HM and bacterial amyloid to protein misfolding and amyloidogenic diseases such as AD are however not well understood, although bacterial components such as endotoxins are often found within the senile plaque lesions that characterize the AD brain (5, 21, 38). The HM further appears to condition host immunity to foreign microbes, including viral infection and xenobiotics, while regulating autoimmune responses that can impact homeostatic metabolic- and neural-signaling functions within the CNS (4, 14, 23, 39). Progressive neurological disorders such as AD have been increasingly linked to altered autoimmune and faulty innate-immune responses (12, 40, 41). An increased incidence of auto-immunity, exposure to pathogens both pre- and postnatally, and findings of antibodies to brain-specific antigens are common in disorders as diverse as anxiety, autism, depression, obsessive–compulsive disorder, schizophrenia, Parkinson’s disease (PD), and AD, together suggesting that differences in exposure and genetic vulnerability toward HM-mediated auto-immunity may be significant determinants of age-related neurological disease course and outcome as humans age (14, 17, 23, 39, 42–46);
(6) secretory products of the GI-tract microbiome and translocation of these signaling molecules via the lymphatic and systemic circulation throughout the CNS are just beginning to be identified. Recent advances in metagenomics, RNA sequencing, metatranscriptomics, metaproteomics, and metabolomics continue to clarify our perceptions of the GI-tract HM and its contribution to health and disease. Just as each individual has a unique “*stoichiometrically proportioned*” composition of microorganisms in their microbiome, individuals appear to be variably sensitive to age-related neurological disorders such as AD through the concept of “*human biochemical individuality*” (11, 16, 47). Importantly, dietary and GI-tract HM manipulation and the emergence of personalized medicine may be poised to revise and modernize our remedial efforts in the clinical management of brain disorders including AD, and the progressive transformation to more favorable clinical outcomes (30, 48, 49).

In summary, the human GI tract is a natural habitat for large, diverse, and host-specific microbial communities including multiple species from the kingdoms of *Archaea*, *Bacteria*, *the Viruses*, and other symbiotic microbiota. How humans co-evolved with these complex microbial ecosystems, and how certain microbial species were specifically selected for mutual symbiotic benefit is of extreme interest when assessing critical HM–host interactions involving food digestion, nutrition supply and uptake, metabolic interactions, protection against pathogens and immune system development, maintenance, and dyshomeostasis in both health and disease. To cite another relevant example, abundant evidence suggests that human mitochondria originated from bacteria via endosymbiotic relationships from very early in the evolutionary history of eukaryotes, so cross-reactivity of mitochondria and host immunological responses to selective bacterial GI constituents may have deleterious effects on human mitochondrial function through molecular mimicry (4, 12, 42). This is evidenced by multiple findings in common autoimmune, inflammation-linked systemic, and neurological disorders including ALS, anxiety, diabetes, epilepsy, metabolic disease, obesity, rheumatic fever, schizophrenia, Sydenham’s chorea, PD, AD, and other age-related pathologies, including transgenic animal models for these diseases (2, 4, 12, 23, 44–46, 50–54).

Lastly, since the early investigations of Koch, Metchnikoff, Pasteur, Von Leeuwenhoek, and others on the microbial basis of pathogenicity and disease transmission, Westernized societies have very successfully reduced the incidence of microbial-borne infectious disease, while an environment of autoimmune, cardiovascular, metabolic, and neuroinflammatory diseases continues to flourish. We have only recently begun to truly appreciate the potential for complex and beneficial contributions of the GI-tract HM to host genetics, phenotype, and the development and course of CNS disease. With advancement in next-generation, high throughput sequencing and metagenomic technologies our further investigations into the complex microbial ecosystems within us should yield novel HM manipulative strategies for both the optimization of our health and the more effective clinical management of human metabolic, neuropsychiatric, and neurological disorders.
